# Isolation and characterization of ZK002, a novel dual function snake venom protein from *Deinagkistrodon acutus* with anti-angiogenic and anti-inflammatory properties

**DOI:** 10.3389/fphar.2023.1227962

**Published:** 2023-09-29

**Authors:** Brandon Dow Chan, Wing-Yan Wong, Magnolia Muk-Lan Lee, Patrick Ying-Kit Yue, Xiangrong Dai, Karl Wah-Keung Tsim, Wen-Luan Wendy Hsiao, Mandy Li, Xiao-Yi Li, William Chi-Shing Tai

**Affiliations:** ^1^ Department of Applied Biology and Chemical Technology, The Hong Kong Polytechnic University, Kowloon, Hong Kong SAR, China; ^2^ Department of Food Science and Nutrition, The Hong Kong Polytechnic University, Kowloon, Hong Kong SAR, China; ^3^ Department of Biology, Hong Kong Baptist University, Kowloon, Hong Kong SAR, China; ^4^ Lee’s Pharmaceutical (HK) Limited, Hong Kong Science Park, Shatin, Hong Kong SAR, China; ^5^ Division of Life Science and Center for Chinese Medicine, The Hong Kong University of Science and Technology, Kowloon, Hong Kong SAR, China; ^6^ State Key Laboratory of Quality Research in Chinese Medicine, Macau University of Science and Technology, Taipa, Hong Kong SAR, China; ^7^ Zhaoke (Hong Kong) Ophthalmology Pharmaceutical Limited, Hong Kong Science Park, Shatin, Hong Kong SAR, China; ^8^ State Key Laboratory of Chinese Medicine and Molecular Pharmacology (Incubation), Shenzhen Research Institute of the Hong Kong Polytechnic University, Shenzhen, Hong Kong SAR, China

**Keywords:** ZK002, snake venom protein, anti-angiogenesis, anti-inflammation, dual-function

## Abstract

**Introduction:** Pathological angiogenesis, the abnormal or excessive generation of blood vessels, plays an important role in many diseases including cancer, diabetic retinopathy, psoriasis, and arthritis. Additionally, increasing evidence supports the close linkage between angiogenesis and inflammation. Snake venoms are a rich natural source of biologically active molecules and carry rich potential for the discovery of anti-angiogenic and anti-inflammatory modulators.

**Methods:** Here, we isolated and purified a novel protein, ZK002, from the venom of the snake *Deinagkistrodon acutus*, and investigated its anti-angiogenic and anti-inflammatory activities and mechanisms.

**Results:** ZK002 was identified as a 30 kDa heterodimeric protein of α and β chains, which exhibited anti-angiogenic activity in various *in vitro* assays. Mechanistically, ZK002 inhibited activation of VEGF signaling and related mediators including eNOS, p38, LIMK, and HSP27. ZK002 also upregulated the metalloproteinase inhibitor TIMP3 and inhibited components of the VEGF-induced signaling cascade, PPP3R2 and SH2D2A. The anti-angiogenic activity of ZK002 was confirmed in multiple *in vivo* models. ZK002 could also inhibit the *in vitro* expression of pro-inflammatory cytokines, as well as *in vivo* inflammation in the carrageenin-induced edema rat model.

**Conclusion:** Our findings highlight the potential for further development of ZK002 as a dual function therapeutic against diseases with involvement of pathogenic angiogenesis and chronic inflammation.

## 1 Introduction

Pathological angiogenesis, referring to the abnormal or excessive generation of blood vessels, has been shown to play an important role in many diseases including cancer, diabetic retinopathy, psoriasis, and arthritis (Carmeliet, 2003). A host of growth factors, cytokines, and lipid mediators can stimulate angiogenesis, most notably via vascular endothelial growth factor (VEGF) signaling, which is the key regulatory pathway of angiogenesis (Shibuya, 2011). In addition, increasing evidence supports a tight connection between angiogenesis and inflammation. On one hand, during inflammation, immune cells release pro-angiogenic factors, including VEGF, that induce neovascularization. On the other, increased vascularization contributes to the propagation of inflammation by facilitating the migration of immune cells and inflammatory mediators to affected sites (Waldner et al., 2010; Ribatti, 2017). Thus, the two processes are tightly linked, each inducing and advancing the other.

In recent years, there has been an increased focus on the development of anti-angiogenic modulators for the treatment of angiogenesis-related diseases (Ferrara and Adamis, 2016). Snake venoms are a rich natural source for the discovery of biologically active molecules with diverse effects. *Deinagkistrodon acutus,* also known as hundred-pace snake and Chinese moccasin, is a poisonous snake belonging to the viper family and is commonly found in southern China, Taiwan, and northern Vietnam. *D. acutus* has long been used in traditional Chinese medicines for the treatment of arthritis, and thus has significant medical and commercial value. Recent studies have shown that snake venoms contain a mixture of proteins and peptides including snake venom metalloproteinases, C-type lectins, phospholipase A2, and snake venom serine proteases, and possess a wide range of biological activities including anti-thrombosis, haemostasis, analgesia, anti-tumor, and anti-angiogenic effects (Chen et al., 2019; Mohamed Abd El-Aziz et al., 2019). Many anti-angiogenic snake venom molecules and snake venom protein mimetics are being studied for the treatment of various diseases (Munawar et al., 2018). In our work, we set out to mine the therapeutic potential of proteins isolated from the venom of *D. acutus*.

In this study, we first isolated and purified a novel protein, ZK002, from the venom of *D. acutus* and through *in vitro* and *in vivo* studies, we demonstrated its potent anti-angiogenic effects. Mechanistic investigations demonstrated that the anti-angiogenic activity of ZK002 potentially involved inhibition of the VEGF signaling pathway, upregulation of metalloproteinase inhibitors, and downregulation of components of VEGF-induced signaling. With the aim of expanding the therapeutic scope of ZK002, its effects in inflammation cell line and animal models were also examined. We found that in addition to its anti-angiogenic properties, ZK002 also exhibited strong anti-inflammatory activity, establishing it as a dual function snake venom protein.

## 2 Materials and methods

### 2.1 Materials

*Deinagkistrodon acutus* venom was purchased from Qimen Venom Supplies (Southern Anhui, China). DEAE Sepharose Fast Flow was from Pharmacia (Uppsala, Sweden) and Sephacryl S-100HR was from Cytiva (MA, United States). Protein-PAK DEAE 8HR column was from Waters (MA, United States). All other chemicals used were of analytical grade.

### 2.2 Preparation of ZK002 monoclonal antibody

Primarily isolated ZK002 protein was subjected to SDS-PAGE analysis. Protein of at least 85% purity was confirmed to possess *in vitro* anti-migration and anti-proliferation activity before being used as the antigen for monoclonal antibody production. 6–12 week-old BALB/c mice were used to generate the antibody. Hybridoma cell production was performed by fusing a spleen cell suspension from the immunized mice with myeloma cells at a 1:5 ratio, using a standard polyethylene glycol (PEG) cell fusion protocol. The established hybridoma cell strain was named 1D9 and hybridoma cells were cultured in serum-free media. Hybridoma cell culture media was applied to a recombinant *Staphylococcus aureus* protein A coupled affinity column and monoclonal antibody recognizing ZK002 (purity ≥90%, SDS-PAGE) was purified.

### 2.3 Primary purification of ZK002

Crude *D. acutus* venom was weighed and dissolved in 0.02 M, pH 8.0 Tris-HCl buffer and centrifuged twice at 4,000 RPM for 10 min, with a buffer change between centrifugations. Supernatant was applied to an equilibrated anion-exchange DEAE-Sepharose Fast Flow (FF) column. The target fraction was identified via ELISA using ZK002 monoclonal antibody, and further purified through stepwise separation via cation-exchange chromatography with an equilibrated CM-Sepharose FF column. The resulting eluent was the primary purified ZK002.

### 2.4 ZK002 isolation by antibody affinity chromatography

ZK002 monoclonal antibodies were coupled with a Sepharose 4B column to produce a ZK002 antibody affinity chromatography column. The primary purified ZK002 was applied to the affinity chromatography column equilibrated with buffer (0.02 M Tris-HCl +0.15 M NaCl, pH 7.6). Target protein was eluted, concentrated, and further loaded onto an equilibrated gel-chromatography S-100 filtration column. The resulting eluent was the highly purified ZK002. The purity and molecular weight of the purified ZK002 protein was determined by MALDI-TOF-MS using an Autoflex III mass spectrometer (Bruker, Germany).

### 2.5 Mass spectrometry analysis and protein identification

ZK002 α and β chains were separated by SDS PAGE and the corresponding gel bands excised. Gel bands were dehydrated in 200 μL ACN at room temperature for 10 min, followed by removal using a SpeedVac Vacuum Concentrator. Peptides were then reduced by addition of 25 µL 200 mM DTT solution and incubation at room temperature for 45 min. Next, alkylation was performed by addition of 4 µL 1 M IAA solution and incubation in the dark at room temperature for 20 min. 671 μL 50 mM ammonium bicarbonate solution was added to dilute the urea concentration in the samples to 1 M. For trypsin digestion, 2 µL of trypsin gold solution was added to the samples and incubated at 37°C overnight. Afterwards, 200 µL MilliQ water and 7 µL 100% TFA were added to the samples. The digested peptide samples were cleaned up using a QASIS HLB 1CC Extraction cartridge with two washes of 0.5% TFA before eluting twice with 70% ACN. The eluates were dried with a SpeedVac. Finally, the samples were resuspended in 100 µL 0.1% FA and transferred to LC vials with inserts for Nano-LCMS analysis.

Proteomics analysis was carried out on an UltiMate™ 3,000 RSLCnano System (Thermo Scientific) and Acclaim™ PepMap™ 100 C18 Column 250 mm (Thermo Scientific) with an Orbitrap Fusion Lumos Mass Spectrometer (Thermo Scientific). The mobile phase included 0.1% formic acid in water as solvent A and 0.1% formic acid in acetonitrile as solvent B. The NanoLC program was set as follows: 0–10 min B 2%; 10–12 min B 2%–6%; 12–47 min B 6%–20%; 47–52 min B 20%–30%; 52–56 min B 30%–90%; 56–61 min B 90%; 61–61.1 min B 90%–2%; 61.1–66 min B 2%. Flow rate was set to 0.3 μL/min, and column temperature was 50°C. MS conditions were as follows: Mode of detection: positive mode; Scan range: 400–1,500 m/z; Ionization Source: NSI; Spray Voltage: Static; Positive ion source voltage: 2,300 V; Ion transfer tube temperature: 300°C. The mass spectrometer was operated in data-dependent acquisition mode.

Protein sequences uploaded to the Mascot Server (Matrix Science) and proteomics MS data was processed by Progenesis QI for Proteomics (Waters) with default settings. The peptide ions were identified by Mascot MS/MS ion search of the Uniprot database (Taxonomy: Chordata) using the following criteria: Peptide charge: 2+, 3+, 4+; Peptide tolerance: 10 Da (13C = 2); MS/MS tolerance: 0.1 Da; Fixed modification: Carbamidomethyl (C); Variable modification: Oxidation (M); Enzyme: No specific; Max missed cleavages: 2; Instrument type: ESI-FTICR. The novel ZK002 α-chain MS proteomics data have been deposited to the ProteomeXchange Consortium via the PRIDE (Perez-Riverol et al., 2022) partner repository with the dataset identifier PXD037093.

### 2.6 Crystallization, data collection, and refinement of ZK002

The ZK002 crystals formed rod-like crystals using the hanging drop vapor diffusion method and a Mosquito robot (TTP LabTech) at 19°C utilizing the precipitants 0.2 M (NH_4_)_2_SO_4_, and 20% polyethylene glycol (PEG) 3,350 in 100 mM Tris pH 7.5. The ZK002 crystals formed in the monoclinic space group *P2,* with two copies per asymmetric unit of (αβ)_2_ heterodimers arranged through 2-fold rotation symmetry. Data collection was performed under cryo conditions (100 K) using a Rigaku RU-H3R X-ray instrument. The crystals diffracted to 2.1 Å resolution and all diffraction images were indexed and integrated using the Mosflm program (Leslie and Powell, 2007), and the integrated reflections were scaled using the SCALA program (Evans, 2006). Structure factor amplitudes were calculated using TRUNCATE (French and Wilson, 1978) from the CCP4 program suite. The structure of ZK002 was solved by molecular replacement with the program PHASER (McCoy, 2007), using the refined structure of the α subunit (PDB code: 1WT9) with 100% sequence identity and the β subunit (PDB code: 3BX4) with 56% identity.

All steps of the atomic refinements were performed with the PHENIX.refine program (Afonine et al., 2012). The model was built into 2mFobs—DFcalc, and mFobs—DFcalc maps using COOT (Emsley and Cowtan, 2004). Refinement movements were accepted only when they produced a decrease in the Rfree value. The model was optimized using PDB_REDO (Joosten et al., 2011), and was evaluated with MOLPROBIDITY (Chen et al., 2010). Details of the data collection and refinement statistics of the ZK002 structure are described in Table 1. The coordinates of ZK002 were deposited in the RCSB Protein Data Bank with accession code 7QAJ.

**TABLE 1:** X-ray data collection statistics for the ZK002 crystal.

	HG1
Data collection	
PDB code	7QAJ
Space group	P 1 2 1
Cell dimensions	
a, b, c (Å)	103.16, 54.29, 107.64
α, β, γ (˚)	90, 100.56, 90
No. of copies in a.u	2
Resolution (Å)	27.45–2.10
Upper resolution shell (Å)	2.17–2.1
Unique reflections	68,733 (6,489)^a^
Completeness (%)	98.07 (94.81)
Average I/σ(I)	41.05 (5.19)
Rpim	0.2113 (0.3453)
CC1/2	0.75 (0.735)
Refinement	
Resolution range (Å)	27.45–2.10
No. of reflections [I/σ(I) > 0]	67,661
No. of reflections in test set	3,269
R-working/R-free	0.1990/0.2421
No. of protein atoms	8,334
No. of water molecules	550
Overall average B factor (Å^2^)	38.62
Root mean square deviations	
- bond length (Å)	0.004
- bond angle (°)	0.59
Clashscore	3.1
Ramachandran plot	
Most favored (%)	96.35
Additionally allowed (%)	3.35
Disallowed (%)	0.3

^a^ Values in parentheses refer to the data of the corresponding upper-resolution shell.

### 2.7 Cell culture

HUVECs and RAW264.7 murine macrophages were purchased from the American Type Culture Collection (ATCC) (Manassas, United States). HUVECs were cultured on 0.04% gelatin-coated (Sigma, MO, United States) plates in M199 medium supplemented with penicillin/streptomycin (50 U/mL), 2 mM L-glutamine (Invitrogen, CA, United States), 10% fetal bovine serum (FBS), and 25% EGM at 37°C, 5% CO_2_. HUVECs underwent one passage weekly and were used throughout passages 2 to 4. RAW264.7 murine macrophages were maintained in Dulbecco’s Modified Eagle Medium (DMEM) (Life Technologies, United States) supplemented with 10% heat inactivated FBS and penicillin/streptomycin (50 U/mL) at 37°C, 5% CO_2_. Cell lines were regularly tested and confirmed to be free of *mycoplasma* contamination.

### 2.8 Tube formation assay

Matrigel (BD Biosciences) was plated in a 96-well plate at 37°C for 1 h to allow the matrix solution to solidify. 2 × 10^4^ HUVECs in medium only (uninduced), medium containing 2% FBS, or medium containing 2% FBS and 5 μM ZK002 were then pipetted over the matrix solution and incubated at 37°C for 16 h. Capillary tube structures were photographed with a digital camera attached to an inverted light microscope (×40). Quantification was conducted using WimTube image analysis software.

### 2.9 *In vitro* wound-healing assay

HUVECs were seeded in gelatin coated 6-well plates at density of 3 × 10^5^ cells per well for 24 h. Next, an overlapping double cross shape was scraped in each well using a pipette tip and cells were treated with vehicle (negative control), latrunculin, or ZK002 (5 or 10 µM) and incubated at 37°C for 3 or 6 h. Images were taken before and after drug treatment. Cell migration was analyzed by TScratch software by measuring the area void of cells.

### 2.10 Transwell migration assay

HUVECs were seeded at a density of 5 × 10^4^ cells per well onto 8 µm transwell inserts (Costar, Corning, NY, United States). The lower chamber was filled with 500 μL low serum cell culture medium only, or low serum cell culture medium containing VEGF (10 ng/mL). Cells were then treated with 0, 1, or 5 µM ZK002. After 4 h at 37°C, non-migrated cells were removed with cotton buds and migrated cells were fixed with 4% formaldehyde and stained for 10 min with crystal violet. The magnitude of HUVEC migration was evaluated by counting migrated cells in six random high-power (×100) microscope fields.

### 2.11 Transwell invasion assay

The transwell invasion assay was conducted as the transwell migration assay above, except inserts were coated with 50 µL of matrigel and allowed to solidify in a 37°C incubator for 30 min to form a thin gel layer before the seeding of HUVECs.

### 2.12 Analysis of VEGF-associated protein activation

HUVECs were seeded overnight then serum starved for 3 h in culture medium alone or with ZK002 (5 µM), after which VEGF (100 ng/mL) was applied for 0, 2.5, 5, or 10 min. Cell pellets were collected and stored at −80°C before assessment of the phosphorylation of select molecules by Western blotting.

### 2.13 Western blot analysis

Cell pellets were lysed in RIPA buffer (50 mM Tris-HCl, pH 7.4, 150 mM NaCl, 1 mM EDTA, 1% Triton X-100, 1% sodium deoxycholate, 0.1% SDS) containing 1× Complete protease inhibitor (Roche, Germany) and 1× PhosSTOP phosphatase inhibitor (Roche), and then centrifuged to remove debris. Cell lysates (20 µg) were solubilized in Laemmli sample buffer (250 mM Tris-HCl, pH 6.8, 10% w/v SDS, 0.1% w/v bromophenol blue, 50% v/v glycerol, 10% v/v β-mercaptoethanol), electrophoresed through an SDS-PAGE gel and transferred onto PVDF membranes (Bio-Rad, CA, United States). Blots were then blocked in 5% nonfat skim milk in TBS-Tween20 (TBST) buffer (137 mM NaCl, 2.7 mM KCl, 25 mM Tris-HCl, pH 7.5, 0.1% Tween 20) and probed using the following antibodies in 5% BSA in TBST buffer overnight: p-VEGFR2, p-eNOS, p-p38, p-LIMK, p-cofilin, p-Hsp27, p-Erk1/2 and β-actin (Cell Signaling Technology, MA, United States). Expression was quantified using by scanning autoradiogram and densitometry (Quantity One, Bio-Rad).

### 2.14 Quantitative real-time polymerase chain reaction

HUVECs were treated with ZK002 (5 or 10 µM) for 24 h. RAW264.7 cells were pre-treated with or without ZK002 at 1 μM for 24 h, then stimulated with 0.1 μg/mL LPS for another 6 h. To detect changes in gene expression after ZK002 treatment, total RNA was isolated from cells using the E.Z.N.A.^®^ Total RNA Kit I (Omega Bio-Tek, GA, United States) according to manufacturer’s instructions. RNA integrity was assessed using a Nanodrop One spectrophotometer (Thermo Scientific, MA, United States). Single-strand cDNA was synthesized from 1 μg of RNA using SuperScript^®^ VILO™ MasterMix (Thermo Scientific) according to manufacturer’s instructions. The mRNA levels of select genes were assessed by quantitative real-time polymerase chain reaction (qPCR). Primer sequences are listed in Supplementary Table S1. PCR was performed using PowerUp SYBR Green Master Mix (Applied Biosystems, MA, United States). Amplification and detection were performed using a QuantStudio 7 Real-Time PCR system (Applied Biosystems) at the following conditions: 2 min at 50°C, 10 min at 95°C, followed by 45 cycles of 15 s at 95°C, and 1 min at 60°C. Relative gene expression was calculated using the 2^−ΔΔCT^ method. β-actin was used as a reference gene. Experiments were conducted in triplicate.

### 2.15 Chicken embryo chorioallantoic membrane (CAM) assay

VEGF was mixed with various doses of ZK002 (0.3, 0.8, 1.6, 3.3, or 16.4 μM) in 5 respective chicken embryos. Treatment with VEGF alone served as a positive control. An additional treatment (VEGF +1.6 μM ZK002 + excess ZK002 monoclonal antibody) was used to validate the anti-angiogenic effects of ZK002. Angiogenesis was assessed via observation and photography of blood vessel growth in embryos and quantified using IKOSA CAM Assay online software (Annese et al., 2023).

### 2.16 Animals

Male or female BALB/c nude mice (6–8 weeks) and SD rats (∼200–220 g) were purchased from the Animal Centre of The Chinese University of Hong Kong. Animals were kept in individually ventilated cages in a barrier-sustained animal house, air-conditioned at 20°C ± 2°C and humidity at 55% ± 10%, under a 12 h light/dark cycle. Food and water were provided *ad libitum*, and mice were examined daily. All animal experiments were approved by the Animal Subjects Ethics Sub-Committee (ASESC) of The Hong Kong Polytechnic University and conducted in accordance with the Institutional Guidelines and Animal Ordinance of the Department of Health, HK S.A.R.

### 2.17 *In vivo* matrigel plug assay

8-week-old male BALB/c nude mice were subcutaneously injected at the abdominal midline with 0.4 mL matrigel alone, or matrigel supplemented with endothelial growth supplements (bFGF/heparin) with or without 3 µM ZK002. After 7 days, mice were killed, and vessels penetrating the matrigel were photographed. The matrigel plugs were then carefully extracted, with removal of surrounding connective tissue, and analyzed for hemoglobin levels. Plugs were weighed and homogenized for 5–10 min on ice, then supernatant or standard (50 μL) was added to a 96-well plate in duplicate, followed by 50 μL tetramethylbenzidine. The plate was allowed to develop at room temperature for 15–20 min with gentle shaking, and the reaction was stopped by adding 150 μL of 2 N H_2_SO_4_. Absorbance was read at 450 nm, and hemoglobin levels were normalized to the weight of the plugs.

### 2.18 Directed *in vivo* angiogenesis assay (DIVAA)

The DIVAA assay was performed according to manufacturer’s protocols (Trevigen, MD, United States). Briefly, 8 week-old female nude mice were anesthetized and a 1 cm incision was made on both dorsal-lateral surfaces of the animal, into which two basement membrane extract (BME)-filled angioreactors were implanted on each side. Each angioreactor contained a total of 25 μL BME premixed with VEGF, bFGF, and heparin with or without 3 μM ZK002. Angioreactors containing no growth factors or ZK002 served as controls. After 7 days, mice were killed, and the angioreactors photographed and removed. Hemoglobin content was measured as detailed above.

### 2.19 Acute carrageenan-induced inflammation rat model

Male SD rats (200–220 g each) were used for acute carrageenan-induced paw edema inflammation experiments. Animals were divided into 4 groups: uninduced, vehicle, ZK002 (2.5 mg/kg), and indomethacin (positive control; 10 mg/kg). Drugs were administered via intraperitoneal injection (ZK002) or oral administration (indomethacin), 1 h before induction. Swelling was induced by subplantar injection of 100 μL of a 1% (w/v) suspension of carrageenan in the right hind paw. Paw volumes of each rat were measured with a plethysmometer every 2 h for up to 6 h post-induction. Degree of edema was calculated by the ratio a/b, where a = right hind paw volume post-carrageenan induction, and b = right hind paw volume pre-carrageenan induction.

### 2.20 Statistical analysis

Results are expressed as mean ± SD (*in vitro* experiments) or mean ± SEM (*in vivo* experiments) of at least three independent experiments. One-way ANOVA followed by Bonferroni post-test were used for statistical analysis. Values of *p* < 0.05 were considered as statistically significant.

## 3 Results

### 3.1 Isolation and purification of ZK002

**FIGURE 1:**
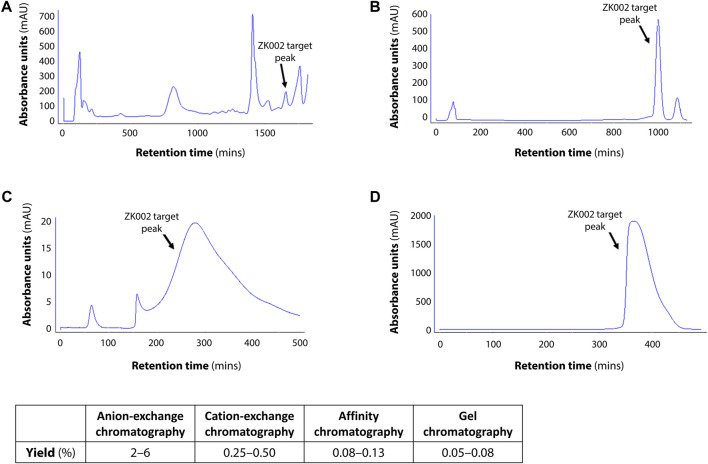
Chromatographic profiles of the purification steps of ZK002. **(A)** Chromatogram of crude solution A from anion-exchange chromatography. **(B)** Chromatogram of crude product B from cation-exchange chromatography. **(C)** Chromatogram of product C from affinity chromatography. **(D)** Chromatogram of high-purity ZK002 from gel chromatography. ZK002 target peaks were identified and confirmed by ELISA with ZK002 monoclonal antibodies. Yields of each step, as a percentage of the initial crude protein amount, are indicated in the table below.

**FIGURE 2:**
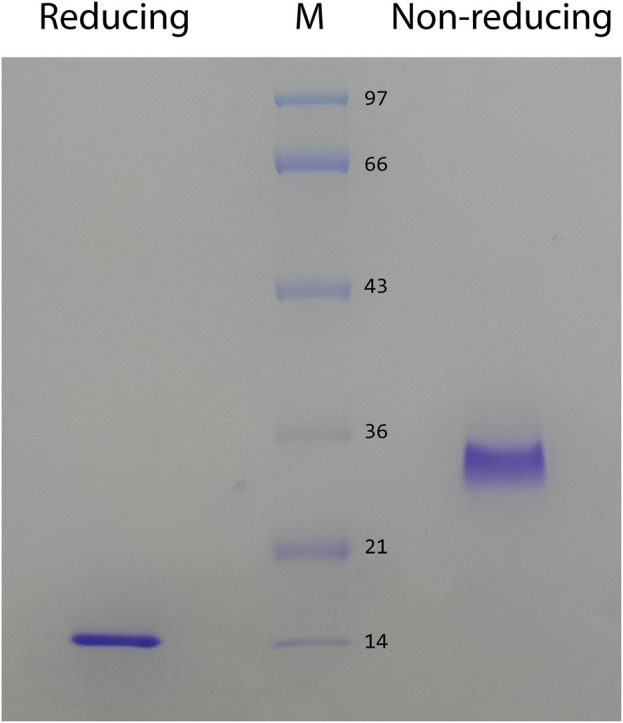
SDS PAGE of the purified protein, ZK002, was conducted under reducing (lane 1) and non-reducing (lane 2) conditions.

In preliminary studies, we found that ZK002 isolated from the venom of *D. acutus* possessed potential anti-angiogenic activity. To facilitate further assessment, a partially purified fraction of ZK002 was extracted from crude *D. acutus* venom via stepwise separation using an anion-exchange column and cation-exchange column. As shown in Figure 1A, six peaks were identified in the eluate from anion-exchange columns, with the ZK002 target peak (identified by ELISA using ZK002 monoclonal antibodies) indicated by an arrow. The collected eluate was further purified by a cation-exchange column (Figure 1B). To obtain highly purified ZK002, the eluate was loaded onto an antibody affinity column (Figure 1C). The fraction was then concentrated on an equilibrated gel-chromatography S-100 column and the resulting eluent was the purified protein (Figure 1D). Gel electrophoresis profiles of the successive fractions indicated the removal of contaminating proteins and enrichment of target (Supplementary Figure S1). The yield of ZK002, as a percentage of total input venom, was 0.05%–0.08%. SDS-PAGE analysis showed a single band for the purified protein, indicating its high purity. Under non-reducing conditions, a band size of approximately 30 kDa was observed, while under reducing conditions, a band of around 15 kDa was seen (Figure 2). These results provided some insight into the structure of ZK002 as a protein of approximately 15 kDa dimers.

**FIGURE 3:**
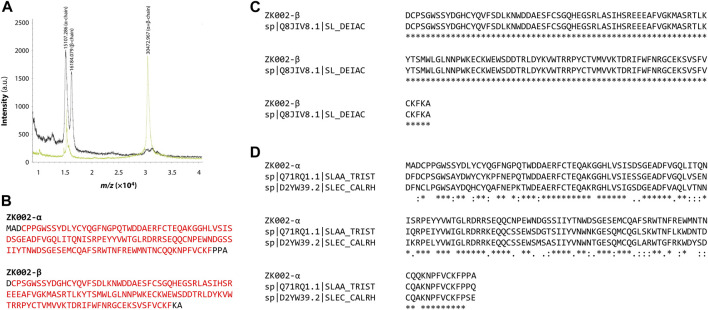
Sequence analysis of ZK002. **(A)** MALDI-TOF mass spectrometry results showing the respective masses of the α and β subunits, and the ZK002 heterodimer. **(B)** Sequence of the identified α and β chains of ZK002. NCBI Conserved Domain Search analysis indicated that both ZK002 α and β possessed C-type lectin-like domains characteristic of snaclecs (matching domain sequences highlighted in red). **(C)** The sequence of ZK002 β shared 100% identity with an existing snaclec (Snaclec clone 2100755, Uniprot Q8JIV8.1). **(D)** The sequence of ZK002 α was aligned with the α chain of stejaggregin-A, and the γ chain of rhodocetin.

By MALDI-TOF mass spectrometry analysis, we identified the mass of purified ZK002 protein as 30,472.967 Da. The purified protein showed a molecular mass of 30 kDa, and two distinct peaks with a molecular mass of 15 kDa (α subunit) and 16 kDa (β subunit) (Figure 3A), results in line with our previous SDS-PAGE experiments indicating that ZK002 is a disulfide-linked heterodimer.

Through tandem mass spectrometry analysis, we identified the protein sequence of ZK002 α and β chains (Figure 3B; Supplementary Figure S2). Database search showed the sequence of the β-chain was a complete match to an existing snake venom C-type lectin protein (snaclec) of *D. actus* (UniProtKB: Q8JIV8). The sequence with highest similarity to the ZK002 α-chain was Snaclec stejaggregin-A subunit alpha (UniProtKB: Q71RQ1.1), to which it shared 76% identity, thus indicating that the α-chain was a novel sequence (Figures 3C, D). NCBI Conserved Domain Database (Marchler-Bauer et al., 2015) analysis indicated that both ZK002 α and β possessed C-type lectin-like domains, a prototypical domain featured in snaclecs.

**FIGURE 4:**
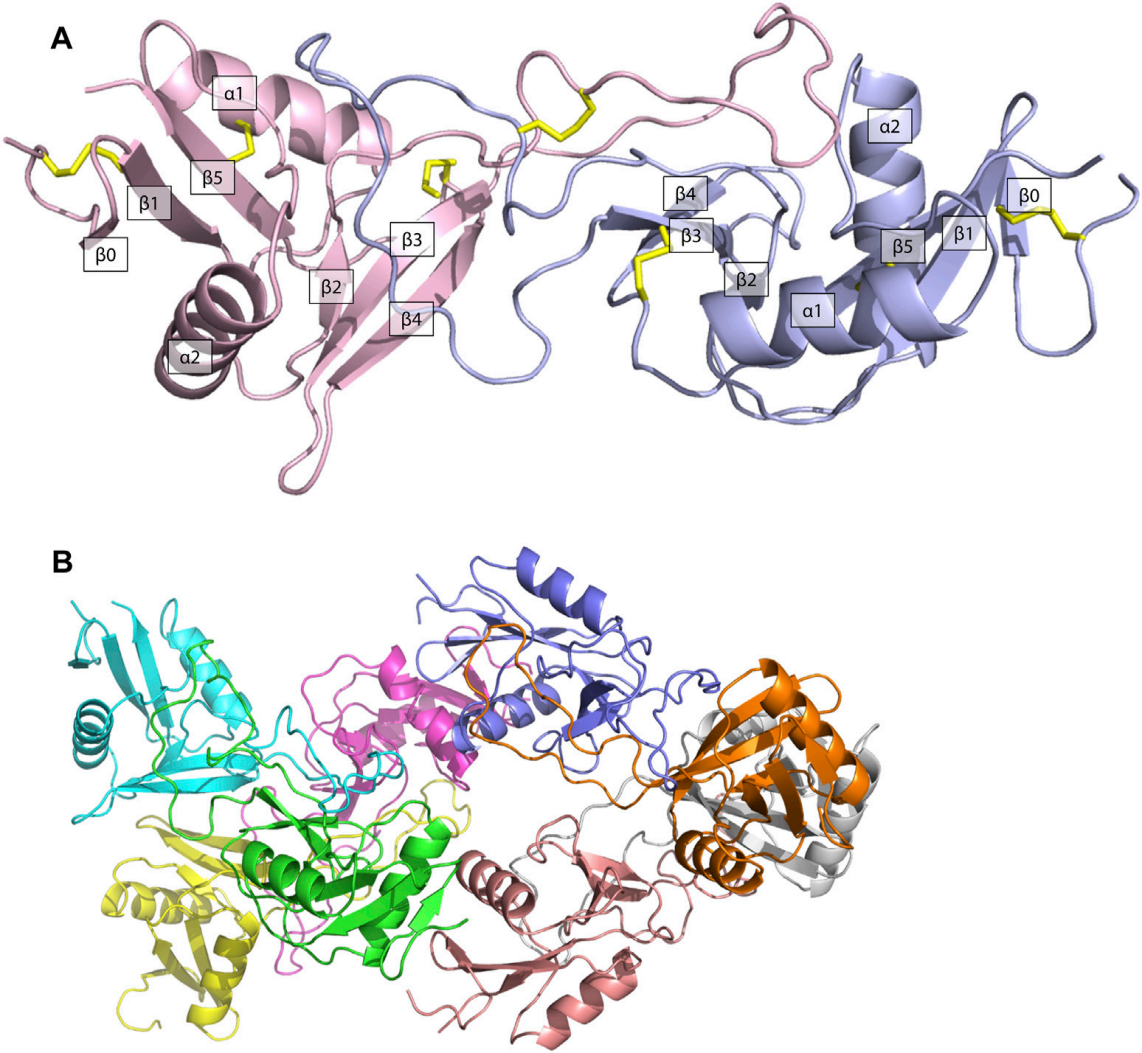
The structure of ZK002 was determined by X-ray crystallography. **(A)** Structure of the αβ chain heterodimer of ZK002. The α chain is displayed in light blue, while the β chain is displayed in pink. Disulphide-bonds formed between cysteine residues are indicated in yellow. **(B)** The crystallographic asymmetric unit of ZK002 containing four αβ heterodimers.

The structure of ZK002 was determined via X-ray crystallography to a resolution of 2.1 Å. In agreement with the mass spectrometry results above, ZK002 exists as an αβ heterodimer. In line with previous reports on snaclecs (Zelensky and Gready, 2005; Eble, 2019), the structure of both α and β chain subunits of ZK002 comprised two β-sheets consisting of the β strands β0–β1–β5 and β2–β3–β4, connected by two orthogonally orientated α-helices, and a long loop was seen between β2 and β3 strands. Further, disulphide bridges between cysteine residues were seen between β0 and β1, α1 and β5, β3 and the loop region of β5, and between the two loop regions of the heterodimers (Figure 4A). The asymmetric crystallographic unit was made up of a tetramer of αβ heterodimers (Figure 4B).

Taken together, the sequence and structure of ZK002 were strongly consistent with that of the snaclecs class of proteins.

### 3.2 ZK002 exhibited anti-angiogenic activity in the HUVEC tube formation assay

**FIGURE 5:**
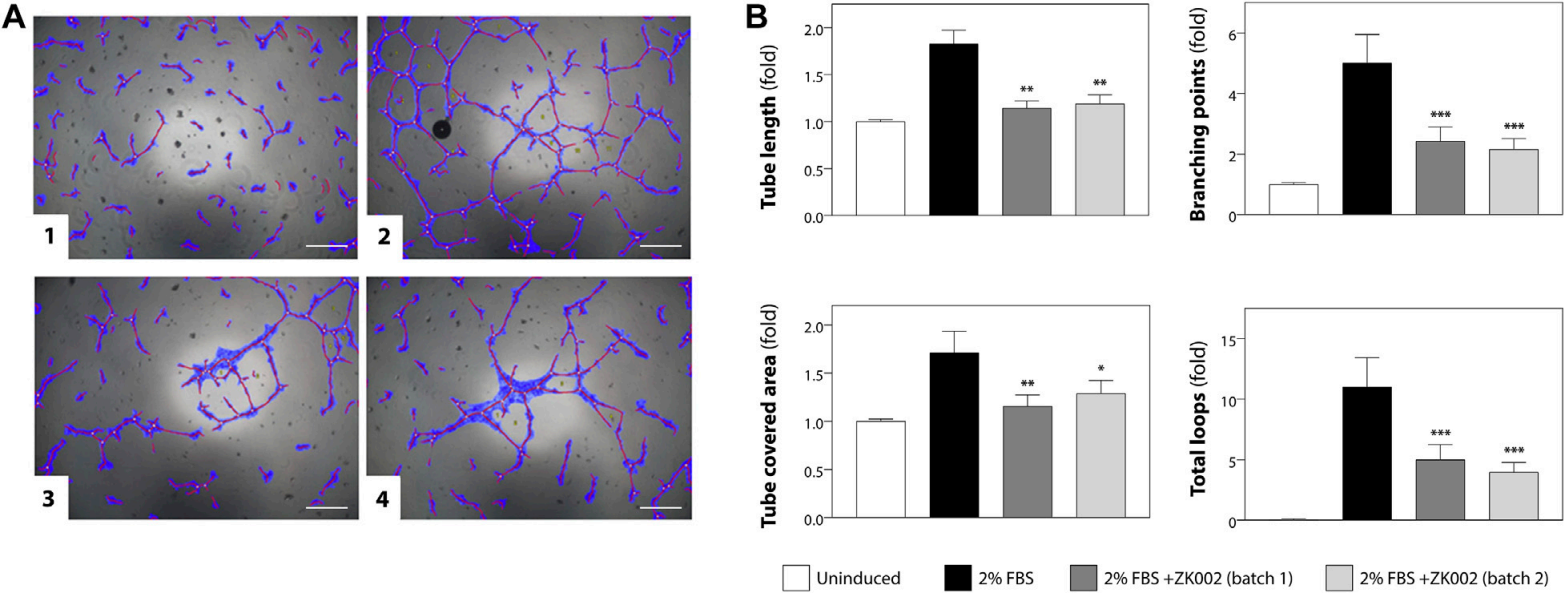
Analysis of anti-angiogenic activity of ZK002 by HUVEC tube formation assay. **(A)** HUVECs (2 × 10^4^) were seeded in 96-well plates containing matrigel, and incubated with 1) Vehicle, 2) 2% FBS, 3) 2% FBS with batch 1 of ZK002 (5 μM), and 4) 2% FBS with batch 2 of ZK002 (5 μM). Capillary tube structures were photographed with a digital camera attached to a microscope (×40 magnification) and analyzed using WimTube software. Scale bar, 200 μm. **(B)** Quantification of the tube formation assays of the four experimental groups. Four aspects of tube formation: i) total tube length, ii) total branching points, iii) total tube covered area, and iv) total loops, were analyzed and scored. *p < 0.05, **p < 0.005, ***p < 0.001 vs. induction control.

The anti-angiogenic activity of ZK002 was first assessed using the HUVEC tube formation assay. Anti-angiogenic activity was quantified based on HUVEC tube length, branching points, tube covered area, and total loops. Figure 5A shows the results of the tube formation assay for two batches of ZK002, of which there was no significant batch-to-batch variation. Both batches of ZK002 displayed comparable anti-angiogenic activity, reducing tube length by an average of 36.2%, branching points by an average of 54.2%, tube coverage by an average of 28.7%, and total loops by an average of 59.3% (Figure 5B).

### 3.3 ZK002 inhibited cell migration and invasion of HUVECs

**FIGURE 6:**
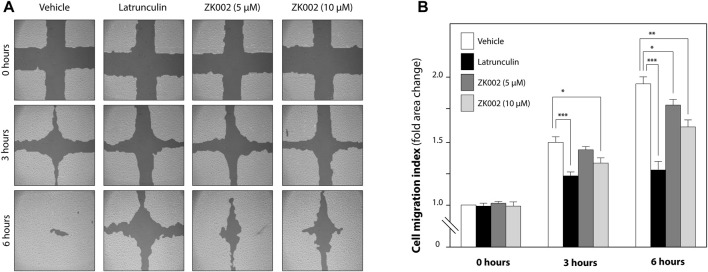
Inhibitory effect of ZK002 on HUVEC cell migration in the *in vitro* wound healing assay. **(A)** Representative photographs of cell migration 0, 3, and 6 h after treatment with ZK002 at 5 or 10 μM. Latrunculin was included as a positive control. **(B)** Cell migration quantification by TScratch software, expressed as cell migration index. *p < 0.05, **p < 0.005, ***p < 0.001 vs. vehicle control.

Based on the above results, we further investigated the *in vitro* anti-angiogenic effects of ZK002. In the wound healing assay, after treatment with ZK002 (5 or 10 µM) for 3 or 6 h, HUVEC migration was analyzed. Results showed that ZK002 could significantly and dose-dependently inhibit the cell migration of HUVECs, up to a 26.4% reduction at 6 h under the 10 µM dose (Figures 6A, B).

**FIGURE 7:**
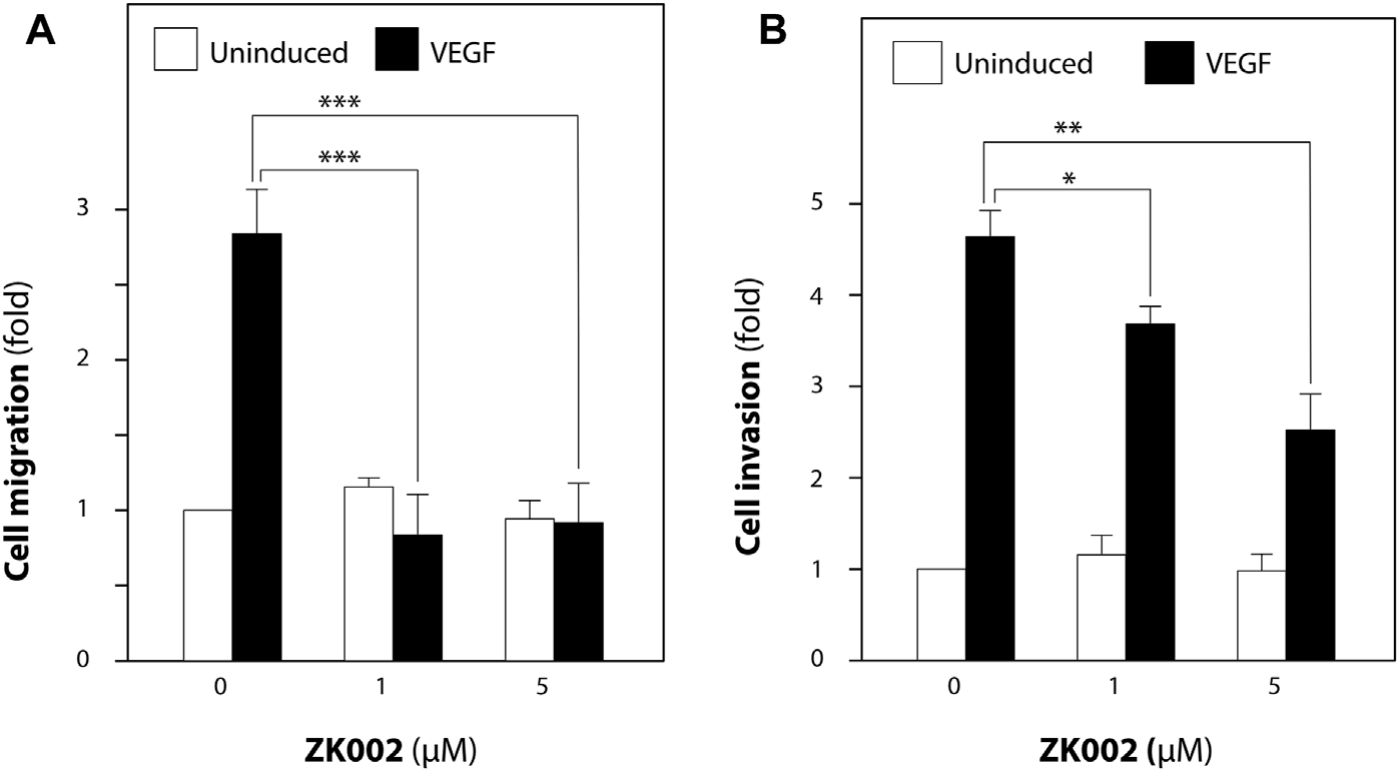
ZK002 inhibited migration and invasion of HUVECs. Effect of ZK002 on VEGF-induced (10 ng/mL) HUVEC migration and invasion in **(A)** Transwell migration assay and **(B)** Transwell invasion assay. *p < 0.05, **p < 0.005, ***p < 0.001 vs. induction control.

We then looked to confirm the anti-cell migratory effect and investigate the anti-invasive effect of ZK002 in VEGF-induced HUVECs via the transwell migration and transwell invasion assays. As shown in Figure 7A, the results confirmed the inhibitory effect of ZK002 on cell migration, completely inhibiting VEGF-induced migration at doses of 1 and 5 µM. Cell invasion was also significantly and dose dependently inhibited by ZK002; there was a 20.5% and 45.6% decrease in invasion of VEGF-induced cells when treated with 1 and 5 µM ZK002 respectively (Figure 7B).

### 3.4 ZK002 exhibited anti-angiogenic effects in the chicken embryo chorioallantoic membrane assay

**FIGURE 8:**
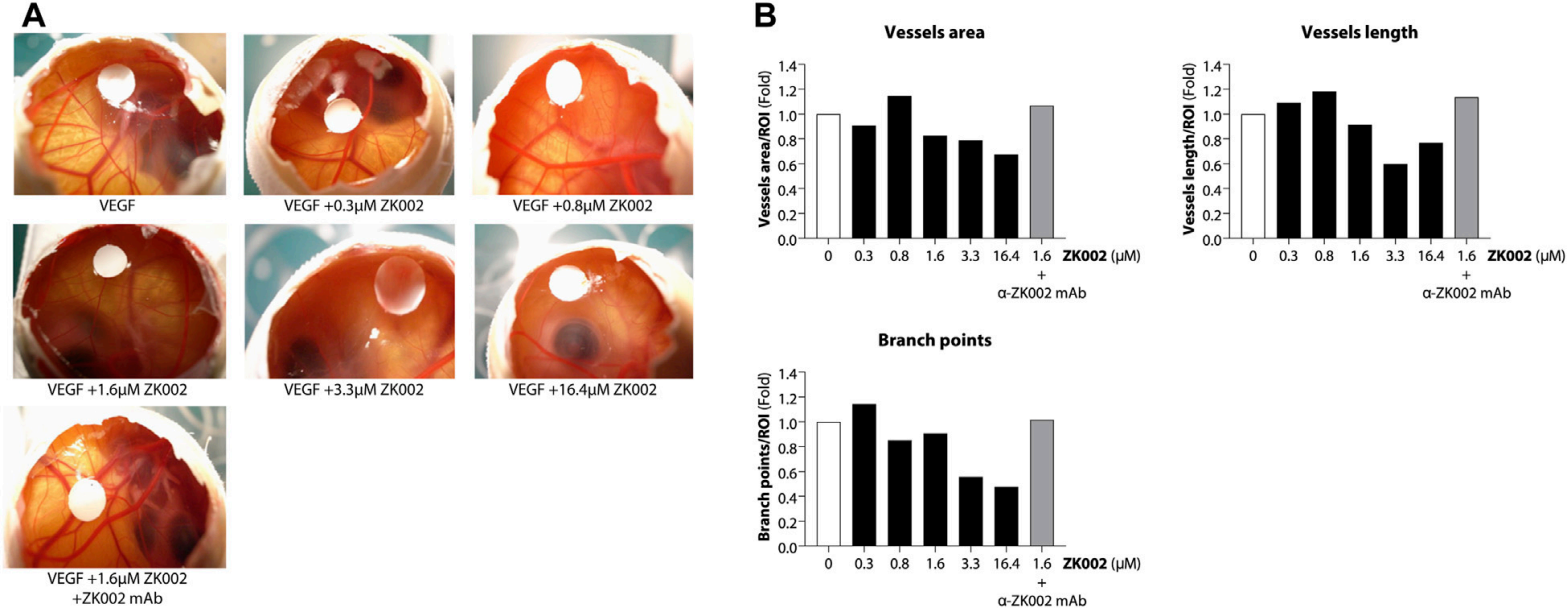
Anti-angiogenic effect of ZK002 in the chicken embryo chorioallantoic membrane (CAM) assay. Chicken embryos were induced with VEGF and treated with ZK002 at various doses from 0.3 to 16.4 μM. A VEGF-induced embryo treated with 1.64 μM ZK002 and an excess amount of anti-ZK002 monoclonal antibody was included as a control. **(A)** Representative photos show blood vessel growth at the experimental endpoint. **(B)** The effect of ZK002 on angiogenesis parameters were assessed using IKOSA CAM Assay software.

After demonstrating the *in vitro* anti-angiogenic efficacy of ZK002, we next examined its *in vivo* anti-angiogenic effects. In the chicken embryo CAM assay, ZK002 dose-dependently suppressed VEGF-induced angiogenesis. At the dose of 16.4 μM, vessel area was decreased by 32.6%, vessel length by 23.3%, and branch points by 52.2% when compared to control (Figures 8A, B). Notably, the ZK002 + ZK002 mAb group showed a similar extent of angiogenesis as the untreated control, confirming that the observed anti-angiogenic effects were due to ZK002 treatment.

### 3.5 *In vivo* anti-angiogeneic effects of ZK002 in the matrigel plug assay and directed *in vivo* angiogenesis assay models

**FIGURE 9:**
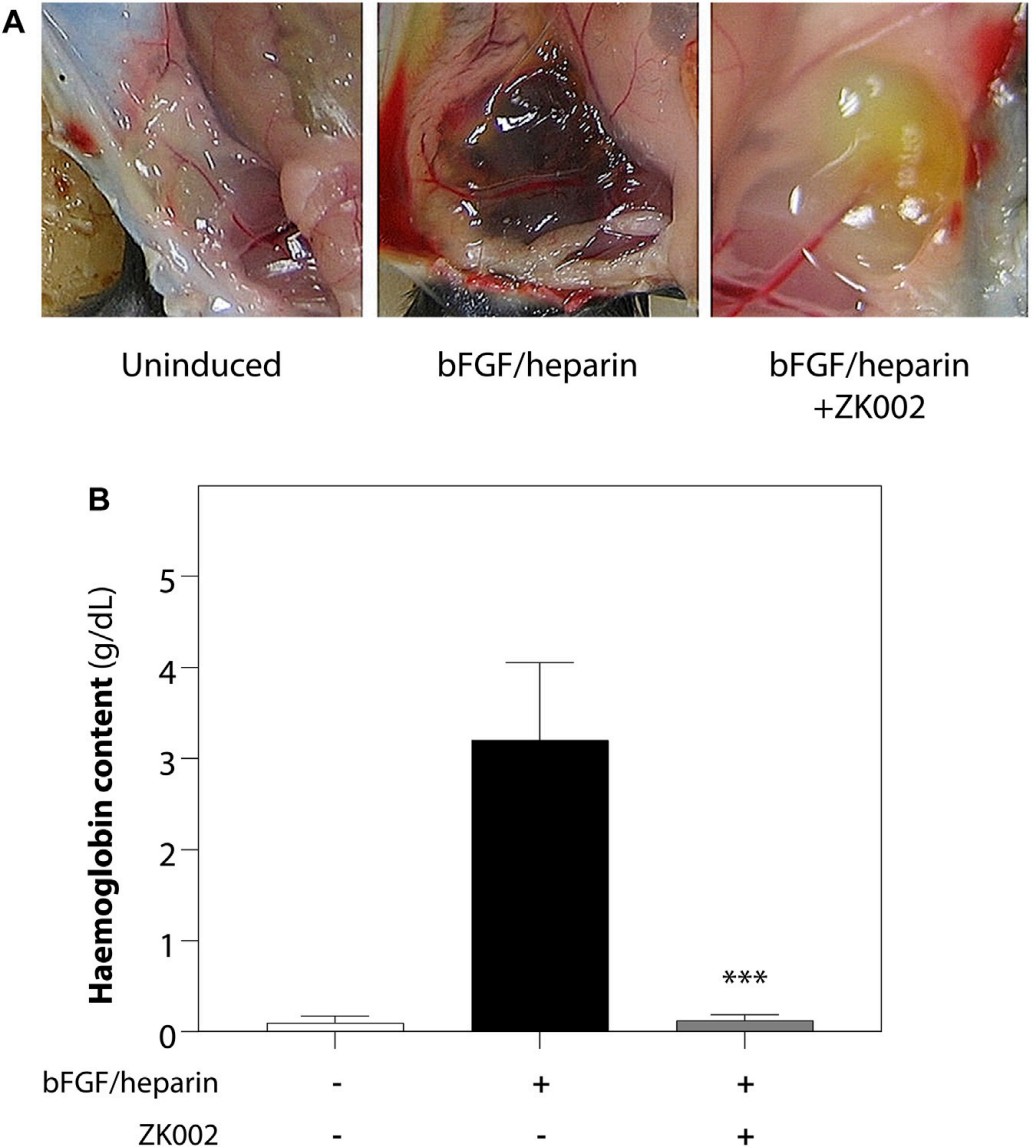
Inhibition of bFGF/heparin-induced *in vivo* angiogenesis by ZK002 in the matrigel plug assay. **(A)** Representative images showing gross morphology of gel plugs in uninduced (matrigel alone), induced (bFGF/heparin), and ZK002 (3 μM) treatment groups. **(B)** Extent of angiogenesis was quantified via measurement of hemoglobin content in matrigel plugs. ***p < 0.001 vs. induced group.

We further investigated the anti-angiogenic effects of ZK002 by performing an *in vivo* matrigel plug assay in mice. 3 μM of ZK002 was mixed with matrigel and endothelial growth supplements (bFGF/heparin) and injected into the abdomens of mice. As shown in Figure 9A, neovascularization was seen in the matrigel plug upon induction with bFGF/heparin. In the presence of ZK002, the matrigel plugs were a light red or light yellow color compared with induction control, and neovascularization was almost completely attenuated. This observation was validated by measuring the hemoglobin content in each matrigel plug. As shown in Figure 9B, treatment with ZK002 dramatically reduced the hemoglobin content by 96.2%.

**FIGURE 10:**
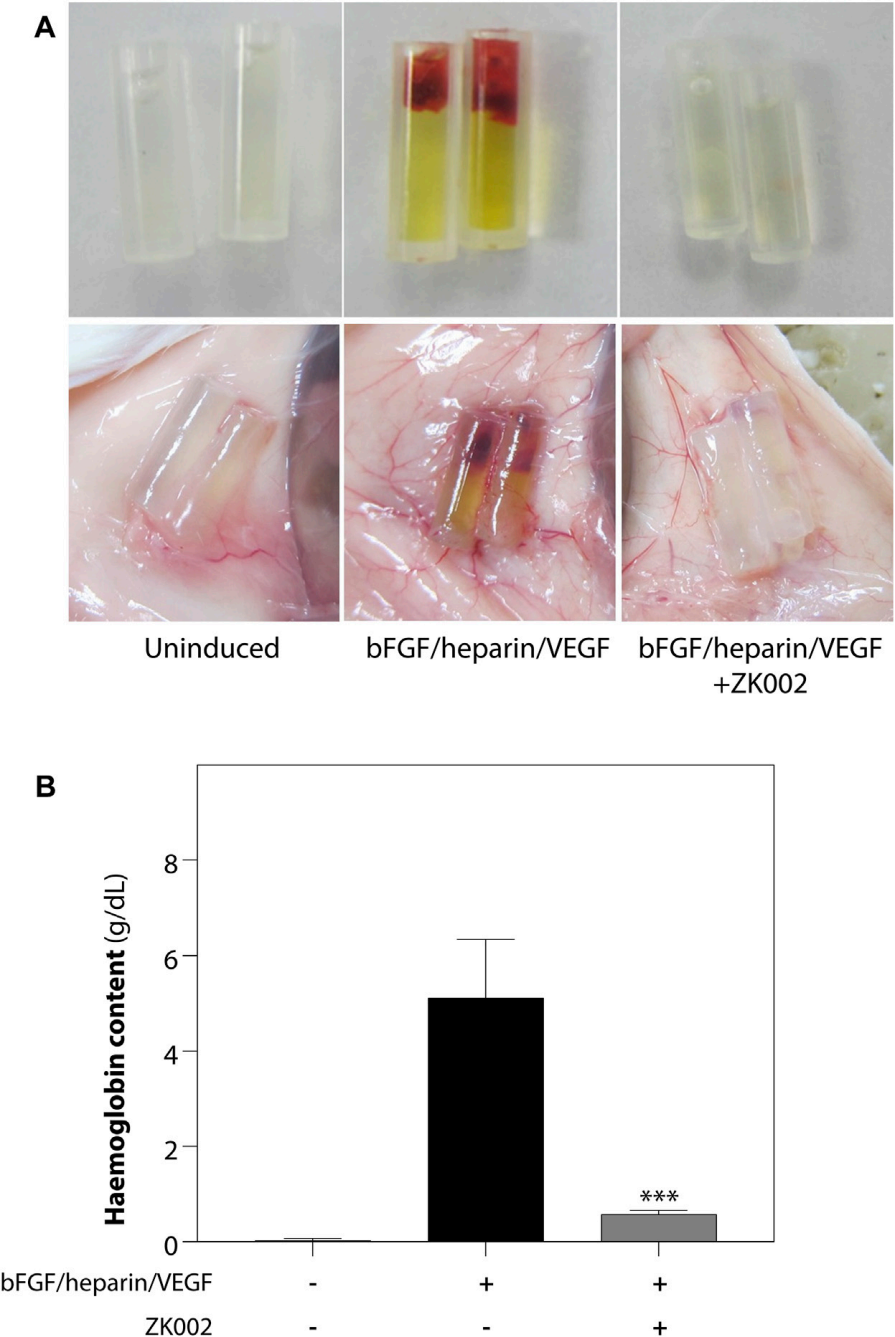
Inhibition of VEGF/bFGF/heparin-induced *in vivo* angiogenesis by ZK002 in the DIVAA assay. **(A)** Representative photographs showing the gross morphology of angioreactors of uninduced (basement membrane extract alone), induced (VEGF/bFGF/heparin), and ZK002 (3 μM) treatment groups. **(B)** Extent of angiogenesis was quantified by measurement of hemoglobin content in angioreactors. ***p < 0.001 vs. induced group.

The above results were confirmed in the DIVAA assay, where in the presence of ZK002, again there was almost complete attenuation of neovascularization. Quantification of hemoglobin content showed an 88.6% reduction in ZK002 treated mice (Figure 10). The above results provide further support for the *in vivo* anti-angiogenic effects of ZK002.

### 3.6 ZK002 inhibited activation of VEGF and VEGF-induced signaling in HUVECs

After demonstrating the *in vitro* and *in vivo* anti-angiogenic activity of ZK002, we investigated the underlying molecular mechanisms involved. As VEGF signaling is the central modulatory pathway in angiogenesis, we examined signaling molecules related to the VEGF signaling cascade and VEGF-induced cell proliferation and migration to see if treatment with ZK002 could affect their regulation.

**FIGURE 11:**
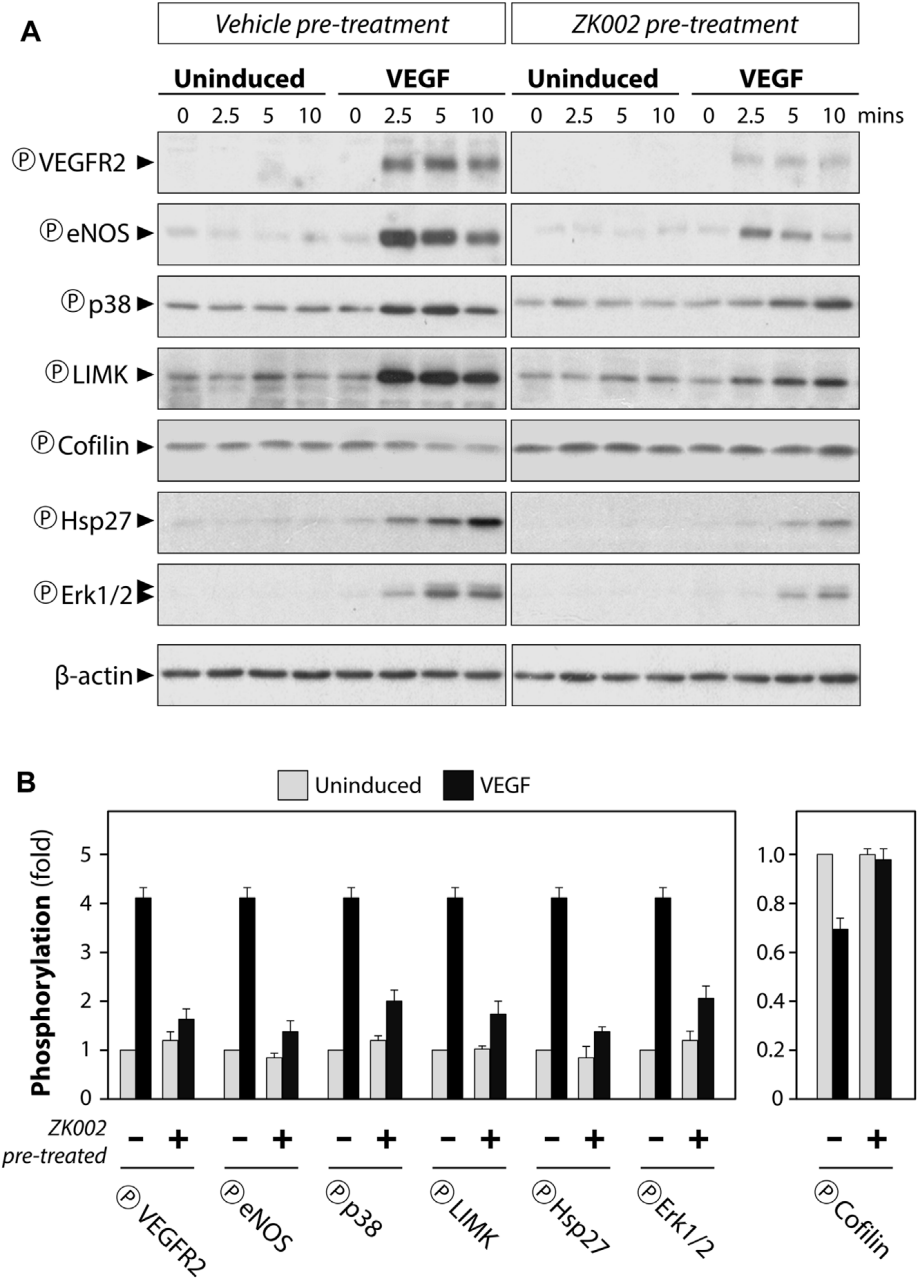
Attenuation of VEGF-induced phosphorylation in HUVECs by ZK002. Serum starved HUVECs were pre-treated with vehicle (water) or 5 µM ZK002 for 3 h, after which they were induced for 0, 2.5, 5, and 10 min with VEGF (100 ng/mL). **(A)** Western blotting results of the phosphorylated forms of select VEGF-related mediators. **(B)** Quantification of band density at the 5 min timepoint was conducted using QuantityOne software analysis from replicate results.

Serum starved HUVECs were pre-treated with vehicle (negative control) or 5 µM ZK002 for 3 h, after which they were induced with VEGF (100 ng/mL) for 0, 2.5, 5, and 10 min. Western blot analysis was carried out to detect the phosphorylation status of select key proteins. Results showed that addition of VEGF indeed induced the phosphorylation of VEGF receptor 2 (VEGFR2), and pre-treatment with ZK002 (5 µM) significantly reduced the VEGF-induced phosphorylation of its receptor (Figures 11A, B). Next, several downstream mediators of VEGF-induced cell migration were examined, including eNOS, p38, LIMK, and HSP27. Pre-treatment with ZK002 could partially reduce the VEGF-induced phosphorylation of the aforementioned molecules. ZK002 also rescued the VEGF-induced decrease in phosphorylation of cofilin, a protein with actin depolymerization activity that is inhibited by LIMK-mediated phosphorylation. We also found that ZK002 treatment could inhibit VEGF-induced phosphorylation of the pro-cell proliferation mediator ERK1/2, suggesting its cell proliferation-inhibitory potential (Figures 11A, B).

Altogether, the above results clearly indicated that ZK002 could block VEGF-related signaling cascades, potentially leading to decreased cell migration and proliferation, and its anti-angiogenic effects.

### 3.7 ZK002 upregulated metalloproteinase inhibitor gene expression and downregulated gene expression of components of VEGF-induced signaling in HUVECs

**FIGURE 12:**
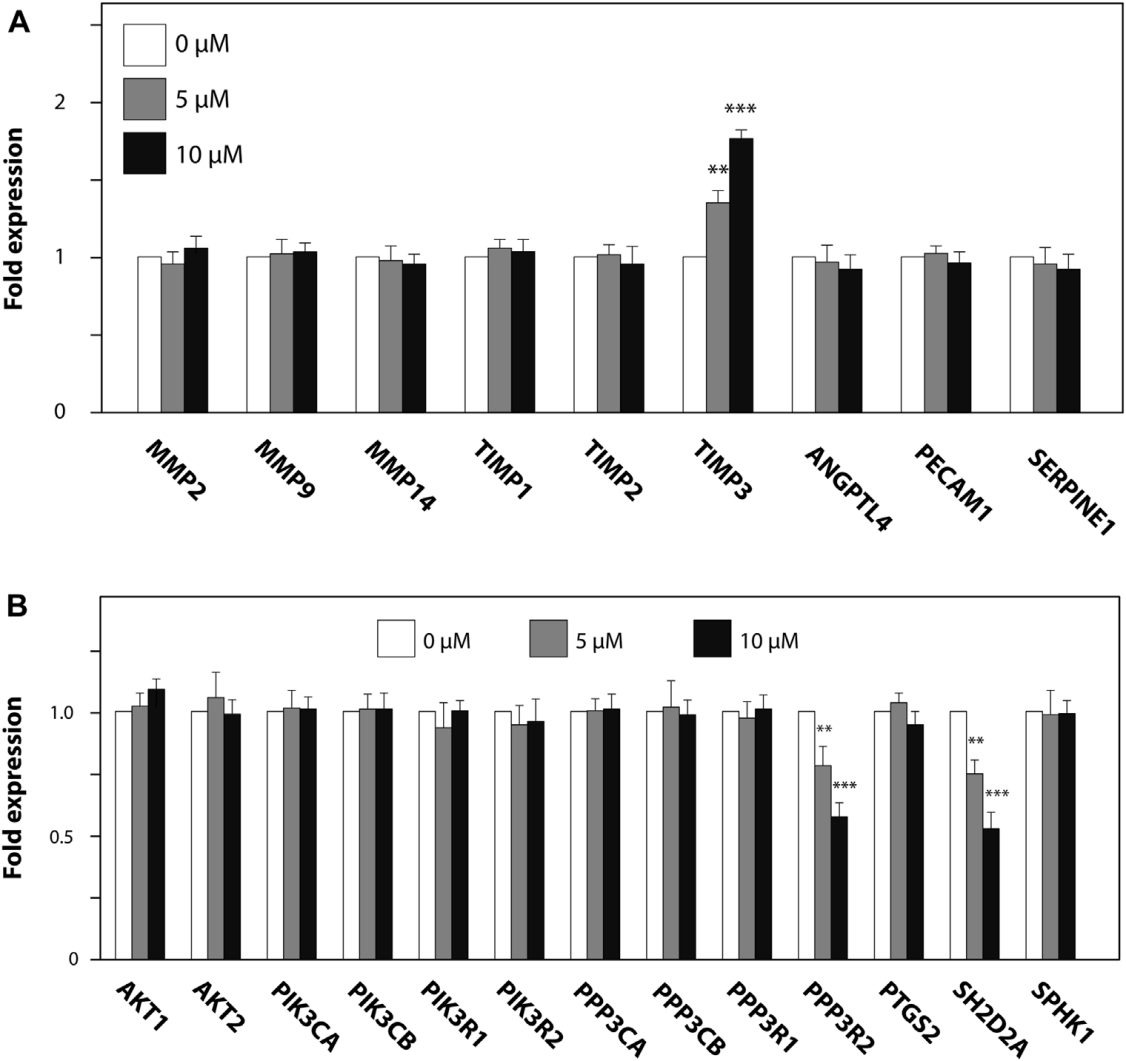
Modulation of metalloproteinase inhibitor and VEGF-induced signaling cascade genes in HUVECs by ZK002. HUVECs were treated for 24 h with 5 or 10 µM ZK002 before collection for qPCR analysis of **(A)** metalloproteinase inhibitor and **(B)** VEGF signaling-related genes. **p < 0.005, ***p < 0.001 vs. untreated control.

We further investigated the effect of ZK002 on the expression of angiogenesis-related genes, whether pro-angiogenic (MMP2/9/14, ANGPTL4, PECAM1, SERPINE1) or anti-angiogenic (TIMP1–3). 24 h of ZK002 treatment in HUVECs significantly and dose-dependently increased expression of the metalloproteinase inhibitor gene TIMP3 when compared to vehicle treated cells (5 μM, 1.34 fold; 10 μM, 1.76 fold; Figure 12A), suggesting that ZK002-mediated inhibition of angiogenesis potentially involved upregulation of metalloproteinase inhibitors. We then continued our analysis, investigating the inhibitory effects of ZK002 on a panel of VEGF-induced signaling cascade genes. We saw that ZK002 treatment could significantly and dose-dependently downregulate the expression of the VEGF-related genes PPP3R2 and SH2D2A (Figure 12B).

### 3.8 ZK002 reduced the gene expression of pro-inflammatory cytokines in LPS-induced macrophages

**FIGURE 13:**
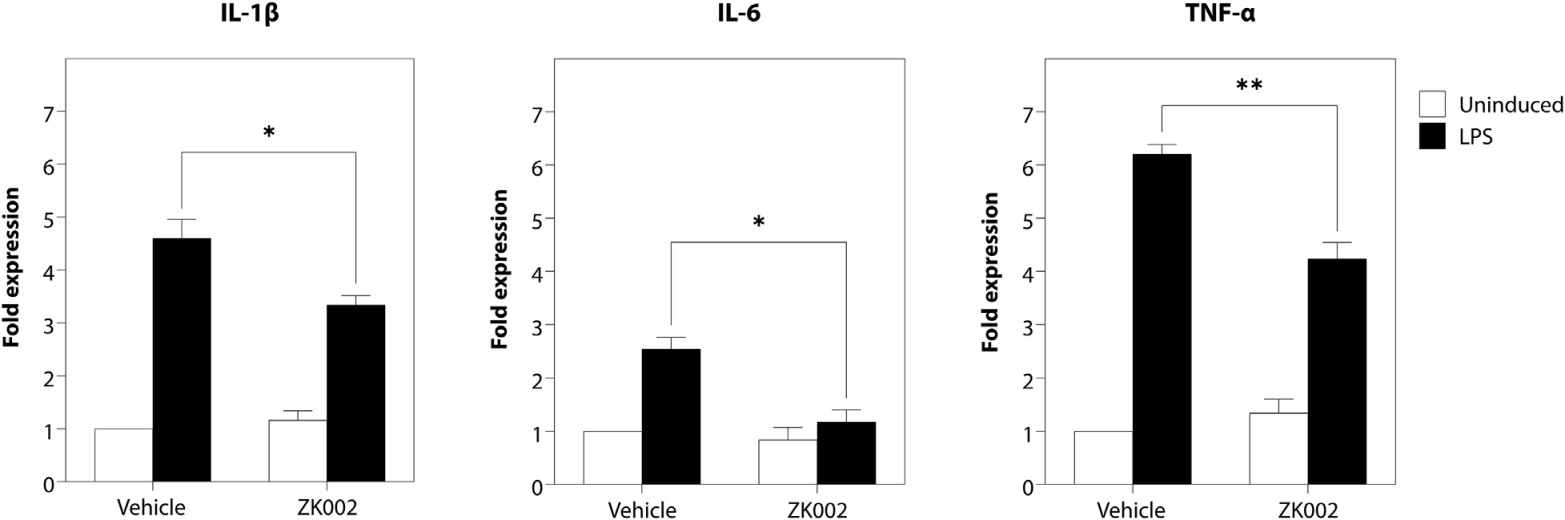
ZK002 inhibited pro-inflammatory cytokine gene expression in LPS-induced RAW264.7 cells. RAW264.7 macrophages (1 × 10^5^) were seeded in each well of a 12 well plate for 24 h. Cells were pre-treated with or without 1 μM ZK002 for 24 h, then stimulated with 0.1 μg/mL LPS for another 6 h. The mRNA levels of IL-1β, IL-6, and TNF-α in cell lysates were measured by qPCR. *p < 0.05, **p < 0.005 vs. vehicle-treated control.

As angiogenesis and inflammation are co-occurrent in many diseases, to investigate whether the scope of the activity of ZK002 could be expanded, we also assessed its potential anti-inflammatory activity. The anti-inflammatory activity of ZK002 was first evaluated by assessment of pro-inflammatory cytokines in LPS-induced RAW264.7 macrophages. Results showed that treatment with ZK002 could significantly decrease gene expression of IL-6, IL-1β, and TNF-α. Notably, 1 μM ZK002 significantly reduced the gene expression levels of LPS-induced IL-6 by 53.8% when compared to vehicle treatment. Additionally, ZK002 significantly reduced the gene expression of IL-1β and TNF-α by 27.5% and 31.8% respectively (Figure 13). These results indicated the *in vitro* anti-inflammatory potential of ZK002.

### 3.9 *In vivo* anti-inflammatory effects of ZK002 in the acute carrageenan-induced inflammation rat model

**FIGURE 14:**
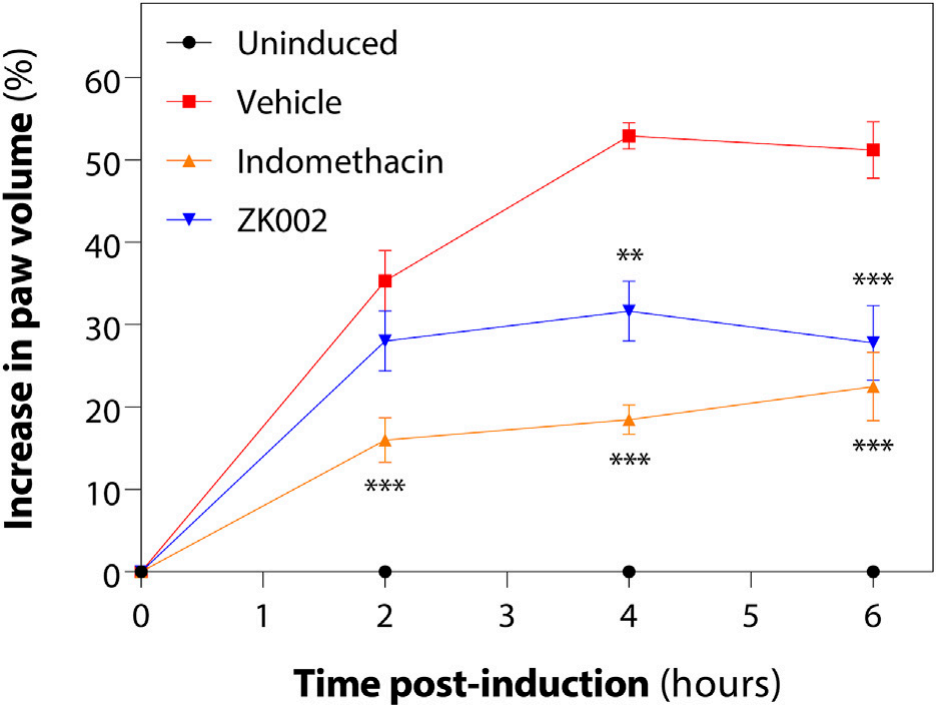
ZK002 inhibited rat paw inflammation in the carrageenan-induced rat model. SD rats were divided into 4 groups: uninduced, vehicle, 10 mg/kg indomethacin, and 2.5 mg/kg ZK002. Drugs were given by intraperitoneal injection (ZK002) or oral administration (indomethacin) 1 h before induction. Edema was induced in hind paws of by subcutaneous injection of carrageenan. Paw volume of each animal was measured with a plethysmometer every 2 h. **p < 0.01, ***p < 0.001 vs. vehicle-treated control.

Upon observing the *in vitro* anti-inflammatory effects of ZK002, we next assessed its *in vivo* anti-inflammatory potential in the acute carrageenan-induced inflammation rat model. Rats were pre-treated with the test compounds for 1 h before induction of edema by injection of hind paws with carrageenan. Paw volume was then measured every 2 h for 6 h in total. At 6 h, we found that 2.5 mg/kg ZK002 could attenuate paw swelling by 46% when compared to vehicle control. This reduction by ZK002 was similar in scale to the positive control, indomethacin, which reduced swelling by 56% but at a comparatively larger dose of 10 mg/kg, indicating the potent anti-inflammatory ability of ZK002 (Figure 14).

Based on these results we identified ZK002 as a dual function protein, exhibiting anti-angiogenic and anti-inflammatory activity, *in vitro* and *in vivo*.

## 4 Discussion

As pathogenic angiogenesis plays a key role in a multitude of diseases, development of novel agents with anti-angiogenic activity are much warranted. In this study, we demonstrated the isolation, purification, and characterization of a novel snake venom protein with anti-angiogenic and anti-inflammatory activity, ZK002.

Snake venoms are a rich natural source for the discovery of novel molecules with biological functions. The most well-studied anti-angiogenic snake venom compounds belong to the disintegrin family of proteins. First isolated from the Viperidae family of venomous snakes, disintegrins were initially identified as inhibitors of platelet aggregation, and several anti-thrombotic drugs have been developed using snake venom disintegrins as lead compounds (e.g., Eptifibatide from the snake venom protein Barbourin, and Tirofiban from the snake venom protein Echistatin). In addition to their anti-thrombotic function, further research has also demonstrated the strong anti-angiogenic activity of disintegrins (Calvete et al., 2005; Swenson et al., 2010; Lazarovici et al., 2019). Another group of proteins found in snake venom are snake venom C-type lectin-like proteins, also known as snaclecs (snake C-type lectins). Snaclecs are approximately 30 kDa in mass with heterodimeric structures comprised of homologous α and β subunits linked by a disulfide bond. Various studies have demonstrated the purification and characterization of snaclecs from the venom of *D. acutus*, with a wide range of biological activities including platelet aggregation, inhibition of platelet aggregation, anti-coagulant activity, erythrocyte-targeting, endothelial adhesion, and angiogenesis (Liu et al., 2002; Xu et al., 2004; Li et al., 2005; Wang, 2008; Zhang et al., 2012). In other studies, snaclecs have been demonstrated to exert anti-angiogenic effects through their modulation of platelet agglutination and coagulation activities (Yeh et al., 2000; Morita, 2005).

In the first part of this paper, we described the isolation and purification of ZK002 and its identification as a 30 kDa heterodimer of α and β peptide chains of the snaclec class of proteins. In contrast to the classical C-type lectins, to which they share sequence homology, snaclecs lack carbohydrate and Ca^2+^ binding abilities and lectin activity. Snaclecs have been shown to act on membrane receptors, coagulation factors, and proteins essential to hemostasis to carry out anticoagulant and antithrombotic activities (Arlinghaus and Eble, 2012). Previous studies have demonstrated that snaclecs, including EMS16 from *Echis multisquamatus*, and Vixapatin from *Vipera xantina palestinae*, exhibit anti-angiogenic effects (Marcinkiewicz et al., 2000; Momic et al., 2012; Chung et al., 2017), indicating the potential for this class of protein as anti-angiogenic drugs.

Our results showed that ZK002 exhibited potent anti-angiogenic activity, with inhibition of tube formation, cell migration, and cell invasion in VEGF-induced HUVECs, and significant anti-angiogenic activity in multiple *in vivo* models. The VEGF proteins are key mediators of angiogenesis, with VEGF-A the prototype member of a family that also includes VEGF-B, VEGF-C, VEGF-D, VEGF-E, and PlGF. VEGF-A binds to two tyrosine kinase receptors, VEGFR-1 and VEGFR-2, VEGFR-2 being the major receptor involved in angiogenic signaling. During angiogenesis, activation of VEGFR-2 promotes cell proliferation, migration, and vascular permeabilization (Apte et al., 2019). In our study, we found that ZK002 treatment inhibited activation of VEGFR2, an event located at the proximal point of VEGF-mediated signaling (Abhinand et al., 2016; Abhinand et al., 2023).

As various studies have shown that VEGF-mediated signaling can trigger activation of MAPK, PI3K/AKT, and eNOS/NO pathways (Koch and Claesson-Welsh, 2012; Yang and Li, 2018), we examined the effects of ZK002 on key molecules of these pathways. Our results showed that ZK002 inhibited the activation of eNOS, p38, HSP27, and LIMK/cofilin activity, all of which have been shown to promote cell migration (Lamalice et al., 2007). We also found that ZK002 inhibited ERK1/2, which mediates cell proliferation during angiogenesis (Srinivasan et al., 2009). This inhibition of pathways implicated in VEGF-induced cell migration and proliferation by ZK002 may further contribute to its anti-angiogenic activity.

To further investigate the molecular mechanisms of ZK002, we examined its effect on angiogenesis-related genes, finding upregulated expression of TIMP3 after ZK002 treatment. Members of the matrix metalloproteinase (MMP) family of proteins are key players in the extracellular matrix (ECM) remodeling activity associated with angiogenesis (Rundhaug, 2005; Ma et al., 2020), and tissue inhibitor of metalloproteinases (TIMPs) are natural inhibitors of the MMP protein family. There exist 4 members of the TIMP family, TIMP 1 to 4, each possessing different specificities and efficacies of MMP inhibition and also exhibiting different activities outside of MMP inhibition. TIMP3 is the only TIMP family member with a high affinity for ECM proteoglycans, and it possesses the broadest range of substrates, including not only all MMPs, but also other ECM-remodeling enzymes from the ADAM (a disintegrin and metalloproteinases) and ADAMTS (ADAM with thrombospondin motifs) families (Brew and Nagase, 2010; Fan and Kassiri, 2020). The TIMP3 gene encodes metalloproteinase inhibitor 3, a potent inhibitor of angiogenesis, whose mechanism of action involves direct binding to VEGFR2, thereby blocking the activity of VEGF (Qi et al., 2003)*.* Previous studies have shown that treatment of VEGF-induced HUVECs with TIMP3 could inhibit their proliferation, migration, and invasion, and also tube formation. Additional studies in the chicken CAM assay demonstrated that angiogenesis was also inhibited by TIMP3 treatment (Lafleur et al., 2003). In other work, silencing of TIMP3 increased expression of MMP2 and MMP9 and capillary network formation (Hu et al., 2016). Altogether, these findings highlight the anti-angiogenic activity of TIMP3, which we found was upregulated by ZK002.

We also found that ZK002 inhibited gene expression of components of the VEGF-induced signaling cascade, PPP3R2 and SH2D2A. PPP3R2 encodes calcineurin subunit B type 2, the calcium-binding regulatory subunit of calcineurin, a calcium-dependent serine/threonine phosphatase that plays a major role in coupling calcium signals to cellular responses (Li et al., 2011). Increased VEGF signaling leads to increased cytoplasmic calcium, activating calcineurin to dephosphorylate the nuclear factor of activated T cells (NFAT) family of transcription factors, which have been shown to be critical in VEGF-induced angiogenesis. Calcineurin-activated NFAT can then translocate to the nucleus to initiate expression of pro-angiogenic target genes (Okamura et al., 2000; Zaichuk et al., 2004; Minami et al., 2013). Previous studies have shown that treatment with various calcineurin inhibitors, including Down syndrome critical region-1 (DSCR1), plasma membrane calcium ATPase (PMCA4), and cyclosporin A could inhibit VEGF-induced *in vitro* and *in vivo* angiogenesis, indicating the relevance of calcineurin inhibition in anti-angiogenesis (Rafiee et al., 2004; Baek et al., 2009; Baggott et al., 2014).

SH2D2A encodes T cell specific adapter protein (TSAd) (Dai et al., 2000), which has been shown to be critical for VEGF-A/VEGFR2 mediated angiogenesis in tumors (Matsumoto et al., 2005). TSAd binds to phosphorylated Y951 on VEGFR2 via its SH2 domain and to the SH3 domain of c-Src via its proline-rich domain to regulate actin reorganization and endothelial cell migration. This VEGFR2-TSAd–c-Src pathway was found to be required for regulation of vascular permeability and angiogenic sprouting, indicating its potential for targeting in pathogenic angiogenesis (Matsumoto et al., 2005; Sun et al., 2012; Gordon et al., 2016). Thus, the inhibition of the two pro-angiogenic genes PPP3R2 and SH2D2A by ZK002 provides further insight into the potential mechanism of its anti-angiogenic activity.

Through the above results we demonstrated that ZK002 could inhibit VEGF -induced, and -downstream signaling. On the other hand, findings from our matrigel plug assay showed that ZK002 could also inhibit FGF-induced angiogenesis, indicating that ZK002 may potentially also act via VEGF-independent mechanisms. The specific molecular target of the anti-angiogenic activity of ZK002 remains to be identified; to further our understanding of the molecular mechanism of ZK002, its direct binding partner is an important item that demands additional investigation.

We also investigated the potential anti-inflammatory effects of ZK002. Our *in vitro* assessments showed that ZK002 could inhibit the major proinflammatory cytokines IL-1β, IL-6, and TNF-α, while *in vivo* investigation demonstrated its potent anti-inflammatory activity in the carrageenin-induced inflammation rat model. Whether this anti-inflammatory activity of ZK002 is linked to, or independent, of its anti-angiogenic effect is of interest for exploration in further studies.

In this study, we demonstrated that the snake venom protein ZK002 exhibited dual anti-angiogenic and anti-inflammatory functions. Angiogenesis and inflammation play a key role in the pathogenesis of many diseases such as cancer, diabetic retinopathy, arthritis, atherosclerosis, obesity, etc. (Carmeliet, 2003; Ribatti, 2017). Based on its dual activity identified in this study, ZK002 would be able to target two pathological aspects at once–a strategic advantage for the treatment of these diseases, highlighting its promise for further development. On the other hand, previous studies have shown that snake venom proteins may also exhibit potential unwanted side effects (Yeh et al., 2000; Li et al., 2005). For future development of ZK002 as a dual functional anti-angiogenic and anti-inflammatory therapeutic protein, further research in the areas of its safety and toxicity are required.

## 5 Conclusion

In this study we isolated and purified a novel protein, ZK002, from venom of the snake *D. acutus*. We then demonstrated that this protein possesses anti-angiogenic and anti-inflammatory effects. In cell line studies, we showed that ZK002 could inhibit the migration and invasion of HUVECs. In *in vivo* models, we demonstrated and confirmed the anti-angiogenic activity of ZK002. Studies into the molecular mechanisms of ZK002 identified inhibition of VEGF-induced signaling components related to cell migration and proliferation, including eNOS, p38, HSP27, LIMK/cofilin, and ERK1/2. Gene expression analysis identified upregulation of the anti-angiogenic metalloproteinase gene TIMP3, and downregulation of the pro-angiogenic genes PPP3R2 and SH2D2A. Further, we also showed that ZK002 could decrease expression of the pro-inflammatory cytokines IL-6, IL-1β, and TNF-α, and inhibit inflammation in an animal model.

Altogether, the results of this study highlight the rich potential for further development of ZK002 as a novel anti-angiogenic and anti-inflammatory protein drug for the treatment of diseases with involvement of pathogenic angiogenesis and inflammation.

## Data Availability

The datasets presented in this study can be found in online repositories. The names of the repository/repositories and accession number(s) can be found below: http://www.wwpdb.org/, 7QAJ https://www.ebi.ac.uk/pride/archive/, PXD037093.
