# Supplementary material for: Isolation and characterization of ZK002, a novel dual function snake venom protein from Deinagkistrodon acutus with anti-angiogenic and anti-inflammatory properties
Source: Front Pharmacol. 2023 Sep 29;14:1227962. doi: 10.3389/fphar.2023.1227962 (PMC10570812; doi:10.3389/fphar.2023.1227962)
Supplement: Supplementary file 1 [file DataSheet1.DOCX]

Supplementary Material

Isolation and Characterization of ZK002, A Novel Dual Function Snake Venom Protein from *Deinagkistrodon acutus* with Anti-angiogenic and Anti-inflammatory Properties

Brandon Dow Chan^†^, Wing-Yan Wong^†^, Magnolia Muk-Lan Lee^†^, Patrick Ying-Kit Yue, Xiangrong Dai, Karl Wah-Keung Tsim*, Wen-Luan Wendy Hsiao*, Mandy Li*, Xiao-Yi Li*, and William Chi-Shing Tai*

^†^These authors contributed equally to this work and share first authorship

*** Correspondence:**William Chi-Shing Tai, william-cs.tai@polyu.edu.hk

Xiao-Yi Li, drli@leespharm.com

Mandy Li, mandy.li@zkoph.com

Karl Wah-Keung Tsim, botsim@ust.hk

Wen-Luan Wendy Hsiao, wlhsiao@must.edu.mo

**
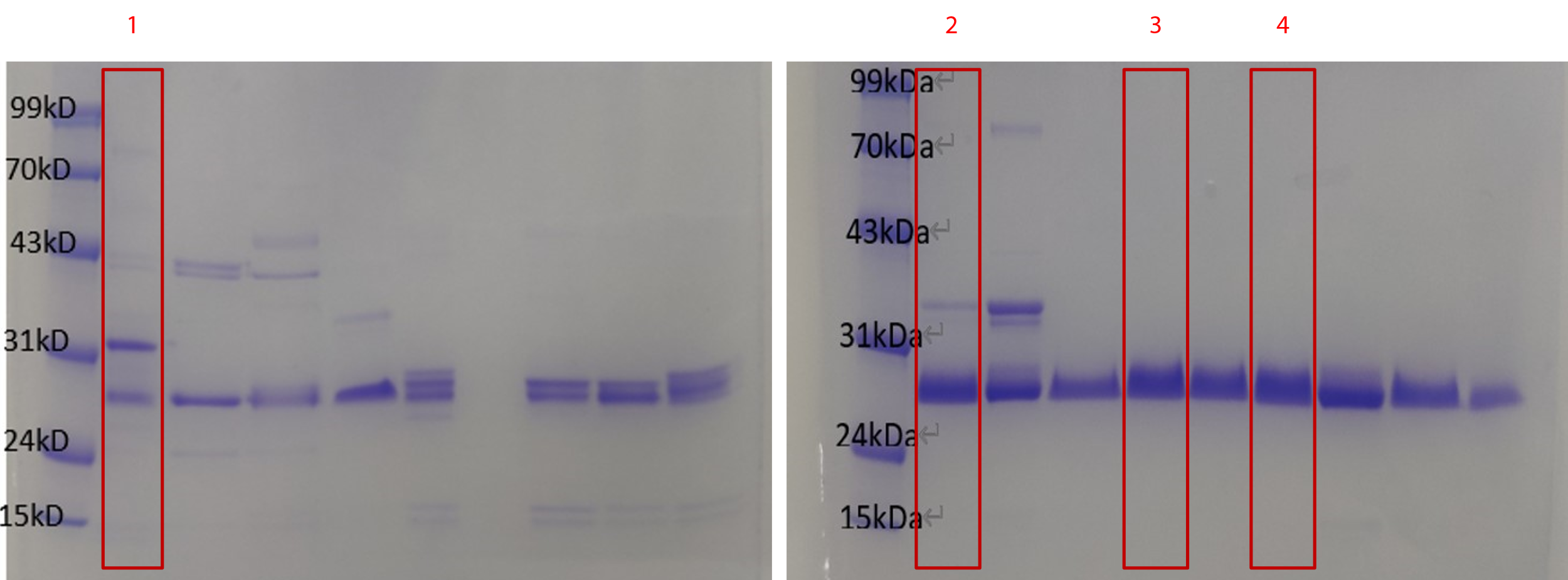
**

**Supplementary Figure 1.** SDS-PAGE (non-reducing) of partially purified fractions was conducted. Fractionated samples are indicated as labelled: (1) Anion-exchange chromatography, (2) Cation-exchange chromatography, (3) Affinity chromatography, (4) Gel chromatography.

**
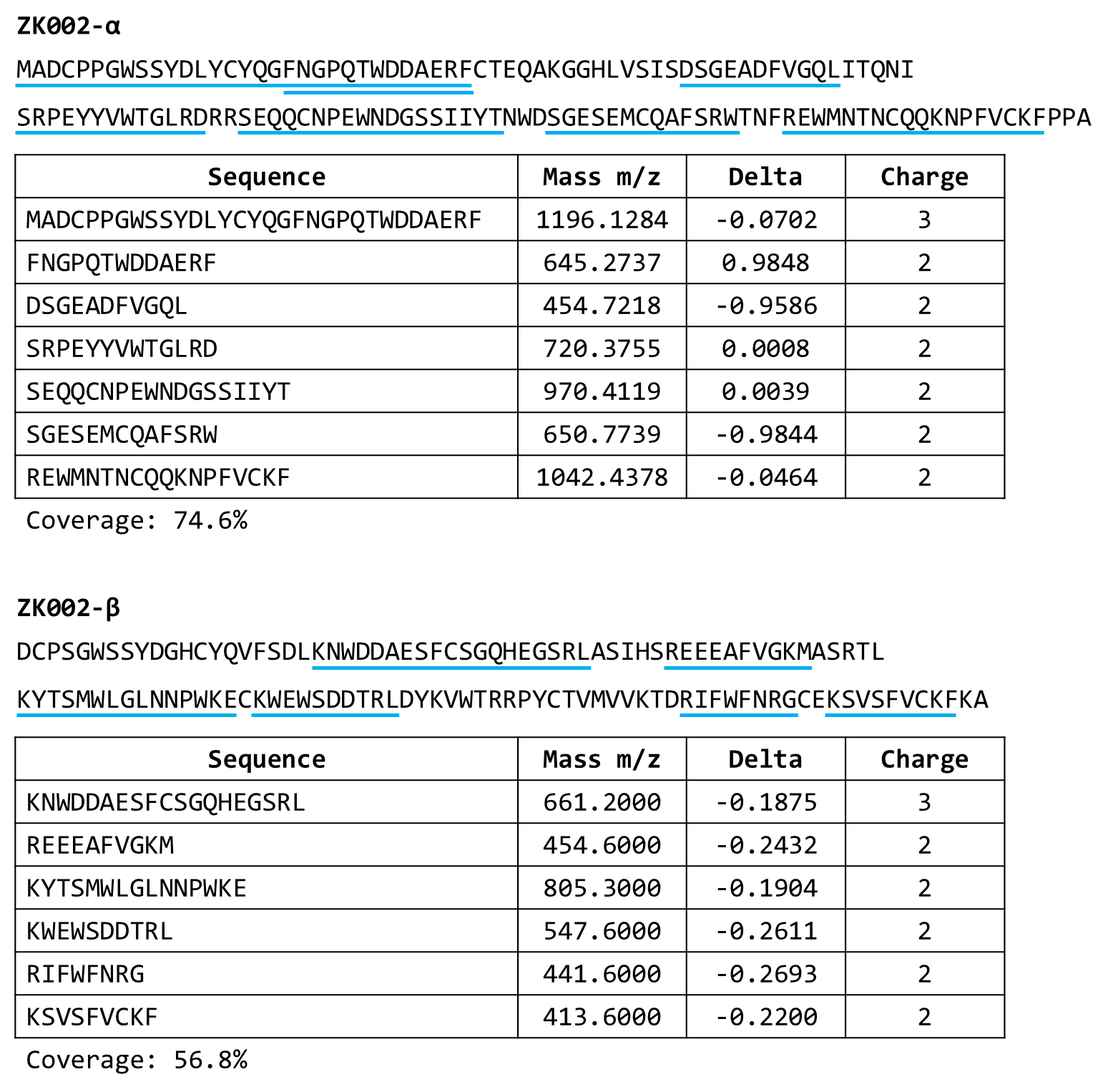
**

**Supplementary Figure 2.** MS/MS analysis of ZK002 α and β chains. Sequences of matching tryptic peptides are underlined in blue.

**Supplementary Table 1.** qPCR Primers used in this study

| **Species** | **Gene** | **Forward primer (5'-3')** | **Reverse primer (5'-3')** |
| --- | --- | --- | --- |
| Mouse | IL-1β | GGAGAACCAAGCAACGACAAAATA | TGGGGAACTCTGCAGACTCAAAC |
| Mouse | IL-6 | CAGAAGGAGTGGCTAAGGACCA | ACGCACTAGGTTTGCCGAGTAG |
| Mouse | TNF-α | TCCCAGGTTCTCTTCAAGGGA | GGTGAGGAGCACGTAGTCGG |
| Mouse | ACTB | GACAGGATGCAGAAGGAGATTACT | TGATCCACATCTGCTGGAAGGT |
|  |  |  |  |
| Human | MMP2 | ATAACCTGGATGCCGTCGT | AGGCACCCTTGAAGAAGTAGC |
| Human | MMP9 | GCCCTTCTACGGCCACTACT | CGTCGAAGATGTTCACGTTG |
| Human | MMP14 | GCCTTGGACTGTCAGGAATG | AGGGGTCACTGGAATGCTC |
| Human | TIMP1 | TGCACAGTGTTTCCCTGTTTATC | GCAGGCAGGCAAGGTGA |
| Human | TIMP2 | ATAAAGATGTTCAAAGGGCCTG | CCTTCTTTCCTCCAACGTC |
| Human | TIMP3 | CGGTATCACCTGGGTTG | GTAGCCAGGGTAACCGAAA |
| Human | ANGPTL4 | CACAGCCTGCAGACACAACTC | GGAGGCCAAACTGGCTTTGC |
| Human | PECAM1 | CTGCTGACCCTTCTGCTCTGTTC | GGCAGGCTCTTCATGTCAACACT |
| Human | SERPINE1 | GACATCCTGGAACTGCCCTA | GGTCATGTTGCCTTTCCAGT |
| Human | AKT1 | CATGAGCGACGTGGCTATTG | GCCTCACGTTGGTCCACATC |
| Human | AKT2 | AAGAAGGCTGGCTCCACAAG | GCATTCTGCTACGGAGAAGT |
| Human | PIK3CA | CCACGACCATCATCAGGTGAA | CCTCACGGAGGCATTCTAAAGT |
| Human | PIK3CB | AGAGCACTTGGTAATCGGAGG | CTTCCCCGGCAGTATGCTTC |
| Human | PIK3R1 | CAGCAACCTGGCAGAATTACGA | TGACAGGATTTGGTAAGTCCAGGAG |
| Human | PIK3R2 | ATGGCACCTTCCTAGTCCGAGA | CTCTGAGAAGCCATAGTGCCCA |
| Human | PPP3CA | GCGCATCTTATGAAGGAGGGA | TGACTGGCGCATCAATATCCA |
| Human | PPP3CB | CCCCAACACATCGCTTGACAT | GGCAGCACCCTCATTGATAATTC |
| Human | PPP3R1 | CCTTTGGAAATGTGCTCACACT | GGATTCTGTTGTAACTCAGGCAG |
| Human | PPP3R2 | GCAGAAGTTGAGGTTTGCGTT | TCTTGTGGATCTCCAGGTCTC |
| Human | PTGS2 | ATATGTTCTCCTGCCTACTGGAA | GCCCTTCACGTTATTGCAGATG |
| Human | SH2D2A | CATCAAACAGGGGCAAGC | GGTGTCGTGGAACAGGGA |
| Human | SPHK1 | AGGCTGAAATCTCCTTCACGC | GTCTCCAGACATGACCACCAG |
| Human | ACTB | CCCTGGACTTCGAGCAAGAGAT | AAGGTAGTTTCGTGGATGCCACA |
